# Crystal structure of bis­(diiso­propyl­ammonium) *cis*-di­iodido­bis­(oxolato-κ^2^ *O* ^1^,*O* ^2^)stannate(IV)

**DOI:** 10.1107/S2056989018003602

**Published:** 2018-03-09

**Authors:** Bougar Sarr, Cheikh Abdoul Khadir Diop, Mamadou Sidibé, Yoann Rousselin

**Affiliations:** a Laboratoire de Chimie Minérale et Analytique, Département de Chimie, Faculté des Sciences et Téchniques, Université Cheikh Anta Diop, Dakar, Senegal; b ICMUB, UMR CNRS 6302, Universite Bourgogne Franche Comte, 9 avenue Alain Savary, 21078 Dijon cedex, France

**Keywords:** crystal structure, tin(IV) oxalate, N—H⋯O hydrogen bonding, bifurcated N—H⋯(O,O) hydrogen bonds

## Abstract

In the title compound, (^i^Pr_2_NH_2_)_2_[SnI_2_(C_2_O_4_)_2_], which was prepared by reacting (^*i*^Pr_2_NH_2_^+^)_2_·C_2_O_4_^2−^ with SnI_4_ in a 2:1 molar ratio in a mixed ethanol–aceto­nitrile solvent, the Sn atom is coordinated by two chelating oxalate ions and two iodide ions, with the latter in a *cis* configuration. In the crystal, the cations are linked to the anions by N—H⋯O and bifurcated N—H⋯(O,O) hydrogen bonds, generating [10-1] chains.

## Abstract 2

The packing of the title mol­ecular salt features N—H⋯O and bifurcated N—H⋯(O,O) hydrogen bonds, which generate [10-1] chains.

## Chemical context   

As a result of their numerous applications (treatment of cancer, fertilizers, PVC stabilizers, catalysts or reaction inter­mediates), organotin compounds have been studied for many years (Christie *et al.*, 1979 ▸; Seik & Kumar Das, 1993 ▸; Ramaswamy *et al.*, 2008 ▸; Reichelt & Reuter, 2014 ▸). As a continuation of our work on organotin compounds (Diop *et al.*, 2002 ▸, 2003 ▸; Sarr *et al.*, 2013 ▸), we now describe the synthesis and crystal structure of the title compound, (I).
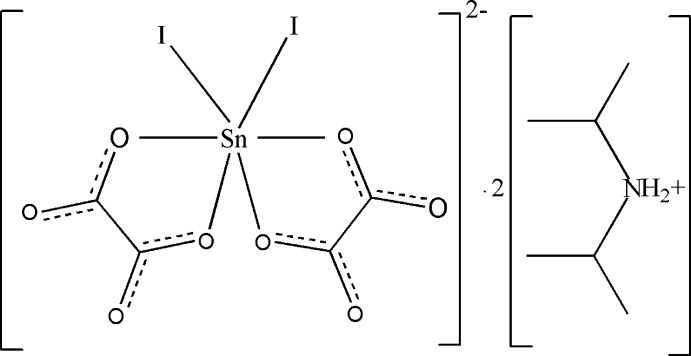


## Structural commentary   

**Figure 1:**
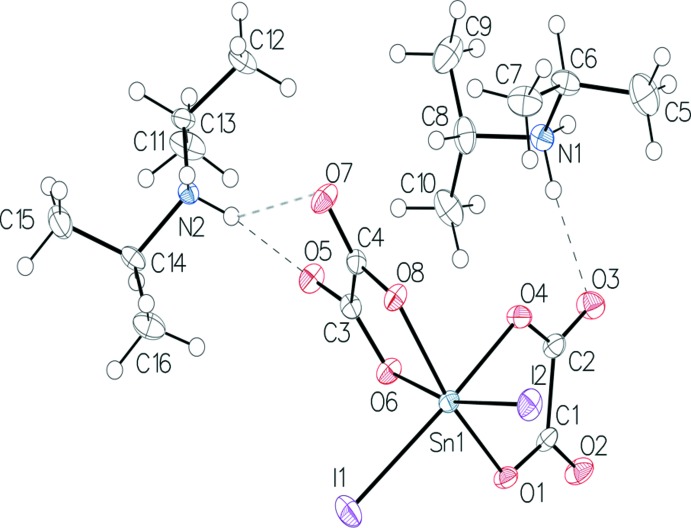
The mol­ecular structure of (I), with displacement ellipsoids depicted at the 50% probability level and N—H⋯O hydrogen bonds shown as dashed lines.

Compound (I) crystallizes in the monoclinic system, space group P2_1_/c with *Z* = 4 formula units. The asymmetric unit contains two diiso­propyl­ammonium cations and one anionic complex [SnI_2_(C_2_O_4_)_2_]^2−^ (Fig. 1 ▸). The Sn^IV^ atom of the stannate anion is six-coordinated by four oxygen atoms arising from two chelating oxalate dianions and two iodo anions in the *cis*-positions, generating a distorted octa­hedral geometry [I2—Sn1—I1 = 99.164 (7), O1—Sn1—O4 = 78.96 (6), O8—Sn1—O6 = 78.60 (5)°]. The C—O bond lengths for the oxygen atoms involved in the coordination of the metal atom [C1—O1 = 1.298 (3), C2—O4 = 1.288 (3), C3—O6 = 1.286 (3), C4—O8 = 1.293 (3) Å] are significantly longer than the non-coordinating C—O bonds [C2—O3 = 1.223 (3), C3—O5 = 1.221 (3), C4—O7 = 1.215 (3), C1—O2 = 1.217 (3) Å]. The Sn—I distances [Sn1—I1 = 2.7190 (2), Sn1—I2 = 2.7039 (2) Å] as well as the Sn1—O distances [Sn1—O1 = 2.0826 (15), Sn1—O4 = 2.1164 (15), Sn1—O6 = 2.1203 (15), Sn1—O8 = 2.0890 (14) Å] are typical and consistent with previous studies (Reichelt & Reuter, 2014 ▸; Skapski *et al.*, 1974 ▸; Sow *et al.*, 2013 ▸). Atoms I1, I2, O4 and O6 are equatorial while O1 and O8 occupy the apical positions in the tin coordination sphere. The angle O1—Sn1—O8 measures 158.49 (6)°: this value deviates considerably from 180°, which may be due to steric hindrance of the iodine atoms. In the equatorial plane the atoms I1, I2, O4 and O6 and the tin(IV) atom are almost coplanar (sum of equatorial angles = 360.3°).

## Supra­molecular features   

**Figure 2:**
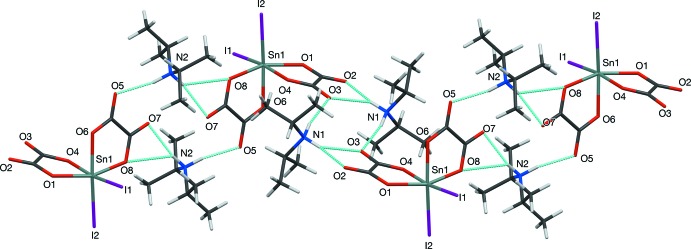
Perspective view of an infinite chain in (I), showing the two types of hydrogen bonds as light-blue dashed lines.

**Figure 3:**
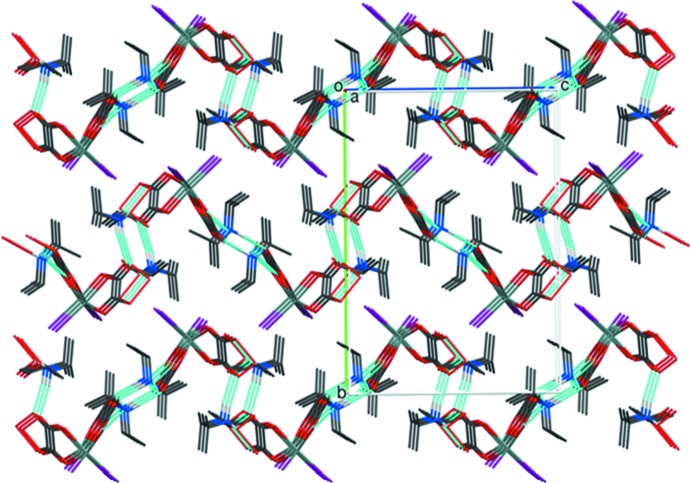
The crystal packing for (I) viewed down [100].

In the crystal of (I), the oxalate ions accept hydrogen bonds from the protonated cations: each cation forms one simple N—H⋯O hydrogen bond and one asymmetric bifurcated N—H⋯(O,O) bond. In the N1 cation, N1—H1*A*⋯(O2^i^,O3^i^) [N⋯O = 2.909 (2), 3.006 (3) Å; symmetry code: (i) −*x* + 1, −*y* + 1, −*z*] and a simple hydrogen bond N1—H1*B*⋯O3 [2.916 (2) Å] (Table 1 ▸); it is notable that O3 accepts a simple and a bifurcated bond. The N2 cation forms a bifurcated N2—H2*B*⋯(O5,O7) bond [2.847 (2), 3.027 (2) Å] and a simple bond N2—H2*A*⋯O7^ii^ [2.968 (2) Å; symmetry code: (ii) −*x* + 2, −*y* + 1, −*z* + 1]. Together, these generate [10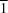] infinite chains as represented in Fig. 2 ▸. The packing also features some weak C—H⋯O inter­actions but the main inter-chain inter­actions are van der Waals forces as shown in Fig. 3 ▸.

## Database survey   

A survey of the Cambridge Structural Database (Version 5.39 plus one update, November 2017; Groom *et al.*, 2016 ▸) reveals 229 hits for diiso­propyl­ammonium [^*i*^Pr_2_NH_2_]^+^ but no hits for the [SnI_2_(C_2_O_4_)_2_]^2−^ anion.

## Synthesis and crystallization   

The title compound was obtained in mixed solvents of ethanol/aceto­nitrile (50/50) by the reaction of bis­(diiso­propyl­ammonium) oxalate (^*i*^Pr_2_NH_2_)_2_·C_2_O_4_ (0.20 g; 0.63 mmol) with tin(IV) iodide (SnI_4_) (0.20 g; 0.32 mmol) in a 2:1 molar ratio. The yellow solution obtained was stirred for 1 h and then filtered. Yellow prisms of (I) were obtained by slow solvent evaporation of the filtrate after two weeks.

The bands at 3039 and 1698 cm^−1^ in the IR spectrum of (I) are assigned respectively to the stretching and deformation vibrations νN—H and δN—H while the broad band at 1676 and those at 1369, 1237 cm^−1^ are attributed to the asymmetric and symmetric vibrations of the oxalate –CO_2_ groups. The shape of the band at 1676 cm^−1^ may be due to a superposition of several bands, which may correlate with the different hydrogen-bonding patterns of the oxalate O atoms. The IR spectrum is available in the supporting information.

## Refinement details   

Crystal data, data collection and structure refinement details are summarized in Table 2 ▸. All H atoms were placed in geometrically idealized positions and constrained to ride on their parent atoms, with C—H distances of 0.98–1.00 Å and an N—H distance of 0.91 Å. All displacement parameters of H atoms *U*_iso_(H) were set to 1.2*U*_eq_(C,N) or 1.5*U*_eq_(Cmeth­yl).

## Supplementary Material

Crystal structure: contains datablock(s) I. DOI: 10.1107/S2056989018003602/hb7738sup1.cif

Structure factors: contains datablock(s) I. DOI: 10.1107/S2056989018003602/hb7738Isup2.hkl

IR spectrum. DOI: 10.1107/S2056989018003602/hb7738sup3.pdf

CCDC reference: 1826865

Additional supporting information:  crystallographic information; 3D view; checkCIF report

## Figures and Tables

**Table 1:** Hydrogen-bond geometry (Å, °)

*D*—H⋯*A*	*D*—H	H⋯*A*	*D*⋯*A*	*D*—H⋯*A*
N1—H1*A*⋯O2^i^	0.91	2.05	2.909 (2)	156
N1—H1*A*⋯O3^i^	0.91	2.37	3.006 (3)	127
N1—H1*B*⋯O3	0.91	2.02	2.916 (2)	168
N2—H2*A*⋯O7^ii^	0.91	2.07	2.968 (2)	167
N2—H2*B*⋯O5	0.91	1.96	2.847 (2)	165
N2—H2*B*⋯O7	0.91	2.48	3.027 (2)	119
C5—H5*C*⋯O2^i^	0.98	2.60	3.359 (3)	135
C13—H13*B*⋯O2^iii^	0.98	2.45	3.397 (3)	162
C14—H14⋯O5^iv^	1.00	2.52	3.508 (3)	170

Symmetry codes: (i) 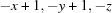; (ii) 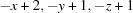; (iii) 
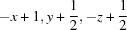
; (iv) 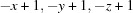.

**Table 2:** Experimental details

Crystal data	
Chemical formula	(C_6_H_16_N)_2_[Sn(C_2_O_4_)_2_I_2_]
*M*_r_	752.92
Crystal system, space group	Monoclinic, *P*2_1_/*c*
Temperature (K)	115
*a*, *b*, *c* (Å)	9.8129 (5), 18.3694 (8), 14.7122 (7)
β (°)	99.769 (2)
*V* (Å^3^)	2613.5 (2)
*Z*	4
Radiation type	Mo *K*α
μ (mm^−1^)	3.38
Crystal size (mm)	0.33 × 0.26 × 0.19

Data collection	
Diffractometer	Nonius Kappa APEXII
Absorption correction	Multi-scan (*SADABS*; Bruker, 2015 ▸)
*T*_min_, *T*_max_	0.601, 0.746
No. of measured, independent and observed [*I* > 2σ(*I*)] reflections	53617, 6000, 5464
*R*_int_	0.025

Refinement	
*R*[*F* ^2^ > 2σ(*F* ^2^)], *wR*(*F* ^2^), *S*	0.018, 0.038, 1.13
No. of reflections	6000
No. of parameters	270
H-atom treatment	H-atom parameters constrained
Δρ_max_, Δρ_min_ (e Å^−3^)	0.53, −0.66

Computer programs: *APEX3* and *SAINT* (Bruker, 2015 ▸), *SHELXT* (Sheldrick, 2015*a* ▸), *SHELXL2018* (Sheldrick, 2015*b* ▸) and *OLEX2* (Dolomanov *et al.*, 2009 ▸).

## supplementary crystallographic information

### Crystal data

**Table:**

(C_6_H_16_N)_2_[Sn(C_2_O_4_)_2_I_2_]	*F*(000) = 1448
*M**_r_* = 752.92	*D*_x_ = 1.914 Mg m^−^^3^
Monoclinic, *P*2_1_/*c*	Mo *K*α radiation, λ = 0.71073 Å
*a* = 9.8129 (5) Å	Cell parameters from 9952 reflections
*b* = 18.3694 (8) Å	θ = 2.2–27.5°
*c* = 14.7122 (7) Å	µ = 3.38 mm^−^^1^
β = 99.769 (2)°	*T* = 115 K
*V* = 2613.5 (2) Å^3^	Prism, clear light yellow
*Z* = 4	0.33 × 0.26 × 0.19 mm

### Data collection

**Table:**

Nonius Kappa APEXII diffractometer	6000 independent reflections
Radiation source: X-ray tube, Siemens KFF Mo 2K-180	5464 reflections with *I* > 2σ(*I*)
Graphite monochromator	*R*_int_ = 0.025
Detector resolution: 512 x 512 pixels mm^-1^	θ_max_ = 27.5°, θ_min_ = 1.8°
φ and ω scans'	*h* = −12→9
Absorption correction: multi-scan (SADABS; Bruker, 2015)	*k* = −23→23
*T*_min_ = 0.601, *T*_max_ = 0.746	*l* = −18→19
53617 measured reflections	

### Refinement

**Table:**

Refinement on *F*^2^	Primary atom site location: structure-invariant direct methods
Least-squares matrix: full	Hydrogen site location: inferred from neighbouring sites
*R*[*F*^2^ > 2σ(*F*^2^)] = 0.018	H-atom parameters constrained
*wR*(*F*^2^) = 0.038	*w* = 1/[σ^2^(*F*_o_^2^) + (0.0083*P*)^2^ + 3.7038*P*] where *P* = (*F*_o_^2^ + 2*F*_c_^2^)/3
*S* = 1.13	(Δ/σ)_max_ = 0.002
6000 reflections	Δρ_max_ = 0.53 e Å^−^^3^
270 parameters	Δρ_min_ = −0.66 e Å^−^^3^
0 restraints	

### Special details

**Table:**

Geometry. All esds (except the esd in the dihedral angle between two l.s. planes) are estimated using the full covariance matrix. The cell esds are taken into account individually in the estimation of esds in distances, angles and torsion angles; correlations between esds in cell parameters are only used when they are defined by crystal symmetry. An approximate (isotropic) treatment of cell esds is used for estimating esds involving l.s. planes.

### Fractional atomic coordinates and isotropic or equivalent isotropic displacement parameters (Å^2^)

**Table:**

	*x*	*y*	*z*	*U*_iso_*/*U*_eq_	
Sn1	0.78573 (2)	0.31450 (2)	0.25379 (2)	0.01392 (4)	
I2	0.99273 (2)	0.25442 (2)	0.17904 (2)	0.02417 (4)	
I1	0.78310 (2)	0.22030 (2)	0.39605 (2)	0.02759 (4)	
O4	0.75849 (16)	0.39502 (8)	0.14940 (11)	0.0192 (3)	
O8	0.92027 (14)	0.39011 (8)	0.32756 (10)	0.0168 (3)	
O6	0.64543 (15)	0.38330 (8)	0.30781 (11)	0.0184 (3)	
O1	0.61143 (16)	0.27511 (8)	0.16703 (10)	0.0185 (3)	
O3	0.59844 (17)	0.43524 (9)	0.03477 (12)	0.0265 (4)	
C3	0.7012 (2)	0.43729 (12)	0.35596 (14)	0.0157 (4)	
C4	0.8615 (2)	0.44251 (12)	0.36505 (14)	0.0160 (4)	
C2	0.6436 (2)	0.38963 (11)	0.09265 (15)	0.0181 (4)	
O5	0.64018 (15)	0.48291 (8)	0.39423 (11)	0.0205 (3)	
O7	0.92302 (16)	0.49320 (9)	0.40632 (11)	0.0237 (4)	
O2	0.44899 (17)	0.31071 (9)	0.05028 (11)	0.0250 (4)	
N2	0.77987 (17)	0.54725 (9)	0.56021 (12)	0.0141 (3)	
H2A	0.873537	0.542656	0.572405	0.017*	
H2B	0.750338	0.526638	0.504073	0.017*	
C11	0.7460 (2)	0.62725 (11)	0.55284 (16)	0.0183 (4)	
H11	0.780729	0.651151	0.613398	0.022*	
C14	0.7209 (2)	0.50374 (12)	0.63123 (15)	0.0179 (4)	
H14	0.617667	0.505053	0.615884	0.022*	
C12	0.8204 (3)	0.65960 (13)	0.47974 (17)	0.0262 (5)	
H12A	0.920144	0.651556	0.497267	0.039*	
H12B	0.801782	0.711982	0.474547	0.039*	
H12C	0.787330	0.636123	0.420273	0.039*	
C13	0.5906 (2)	0.63719 (13)	0.5296 (2)	0.0298 (6)	
H13A	0.554419	0.609123	0.474071	0.045*	
H13B	0.569223	0.688867	0.518454	0.045*	
H13C	0.547803	0.620006	0.581158	0.045*	
C15	0.7659 (3)	0.53661 (15)	0.72612 (17)	0.0293 (5)	
H15A	0.866283	0.544033	0.736978	0.044*	
H15B	0.741206	0.503573	0.773029	0.044*	
H15C	0.719419	0.583483	0.729668	0.044*	
C16	0.7691 (3)	0.42562 (13)	0.62586 (19)	0.0276 (5)	
H16A	0.738383	0.406800	0.563414	0.041*	
H16B	0.729842	0.395753	0.670199	0.041*	
H16C	0.870287	0.423841	0.640488	0.041*	
N1	0.67810 (18)	0.58445 (10)	0.08654 (13)	0.0188 (4)	
H1A	0.616623	0.608623	0.043775	0.023*	
H1B	0.662980	0.535988	0.076743	0.023*	
C6	0.8219 (2)	0.60163 (13)	0.06907 (17)	0.0240 (5)	
H6	0.837304	0.655336	0.075377	0.029*	
C8	0.6461 (2)	0.60273 (14)	0.18069 (16)	0.0260 (5)	
H8	0.722502	0.583589	0.228434	0.031*	
C10	0.5125 (3)	0.56523 (15)	0.19254 (19)	0.0322 (6)	
H10A	0.436853	0.583129	0.145719	0.048*	
H10B	0.492012	0.575786	0.254106	0.048*	
H10C	0.522388	0.512540	0.185450	0.048*	
C9	0.6388 (3)	0.68473 (16)	0.1915 (2)	0.0367 (6)	
H9A	0.728046	0.706354	0.185379	0.055*	
H9B	0.616971	0.696324	0.252461	0.055*	
H9C	0.566489	0.704460	0.143656	0.055*	
C7	0.9297 (2)	0.56314 (14)	0.13818 (19)	0.0304 (6)	
H7A	0.927002	0.582008	0.200145	0.046*	
H7B	1.021584	0.571705	0.122522	0.046*	
H7C	0.910520	0.510758	0.136630	0.046*	
C5	0.8285 (3)	0.57961 (17)	−0.0294 (2)	0.0396 (7)	
H5A	0.816851	0.526780	−0.035890	0.059*	
H5B	0.918410	0.593638	−0.044577	0.059*	
H5C	0.754643	0.604200	−0.071496	0.059*	
C1	0.5582 (2)	0.31967 (11)	0.10212 (15)	0.0174 (4)	

### Atomic displacement parameters (Å^2^)

**Table:**

	*U*^11^	*U*^22^	*U*^33^	*U*^12^	*U*^13^	*U*^23^
Sn1	0.01150 (7)	0.01436 (7)	0.01526 (7)	−0.00066 (5)	0.00046 (5)	−0.00098 (5)
I2	0.02246 (8)	0.02548 (8)	0.02626 (8)	0.00472 (6)	0.00897 (6)	−0.00448 (6)
I1	0.02680 (8)	0.03201 (9)	0.02397 (8)	−0.00116 (7)	0.00428 (6)	0.01047 (6)
O4	0.0185 (8)	0.0158 (7)	0.0215 (8)	−0.0027 (6)	−0.0017 (6)	0.0029 (6)
O8	0.0091 (7)	0.0211 (8)	0.0200 (8)	0.0002 (6)	0.0014 (6)	−0.0063 (6)
O6	0.0096 (7)	0.0216 (8)	0.0235 (8)	−0.0014 (6)	0.0014 (6)	−0.0053 (6)
O1	0.0194 (8)	0.0141 (7)	0.0195 (8)	−0.0032 (6)	−0.0038 (6)	0.0001 (6)
O3	0.0292 (9)	0.0197 (8)	0.0271 (9)	0.0005 (7)	−0.0053 (7)	0.0064 (7)
C3	0.0106 (10)	0.0202 (10)	0.0158 (10)	−0.0007 (8)	0.0012 (8)	0.0020 (8)
C4	0.0119 (10)	0.0220 (11)	0.0145 (10)	−0.0027 (8)	0.0032 (8)	−0.0008 (8)
C2	0.0198 (11)	0.0149 (10)	0.0190 (11)	0.0001 (8)	0.0019 (9)	−0.0021 (8)
O5	0.0128 (7)	0.0242 (8)	0.0248 (8)	0.0016 (6)	0.0038 (6)	−0.0057 (7)
O7	0.0138 (8)	0.0288 (9)	0.0291 (9)	−0.0045 (7)	0.0051 (6)	−0.0135 (7)
O2	0.0223 (8)	0.0205 (8)	0.0277 (9)	−0.0024 (7)	−0.0082 (7)	−0.0018 (7)
N2	0.0100 (8)	0.0167 (9)	0.0159 (9)	−0.0015 (7)	0.0031 (7)	0.0007 (7)
C11	0.0210 (11)	0.0130 (10)	0.0228 (11)	−0.0018 (8)	0.0089 (9)	0.0007 (8)
C14	0.0126 (10)	0.0201 (11)	0.0217 (11)	−0.0006 (8)	0.0048 (8)	0.0069 (9)
C12	0.0276 (13)	0.0232 (12)	0.0300 (13)	−0.0040 (10)	0.0115 (10)	0.0074 (10)
C13	0.0224 (12)	0.0203 (12)	0.0499 (16)	0.0076 (10)	0.0148 (11)	0.0099 (11)
C15	0.0301 (13)	0.0374 (14)	0.0221 (12)	−0.0046 (11)	0.0089 (10)	0.0049 (10)
C16	0.0244 (12)	0.0211 (12)	0.0376 (14)	0.0026 (10)	0.0062 (10)	0.0091 (10)
N1	0.0161 (9)	0.0198 (9)	0.0196 (9)	0.0009 (7)	0.0004 (7)	0.0028 (7)
C6	0.0196 (11)	0.0216 (12)	0.0319 (13)	−0.0025 (9)	0.0073 (10)	0.0039 (10)
C8	0.0207 (12)	0.0389 (14)	0.0172 (11)	0.0048 (10)	−0.0005 (9)	0.0028 (10)
C10	0.0293 (14)	0.0379 (15)	0.0322 (14)	0.0070 (11)	0.0126 (11)	0.0109 (11)
C9	0.0291 (14)	0.0429 (16)	0.0372 (15)	−0.0037 (12)	0.0034 (11)	−0.0158 (13)
C7	0.0165 (12)	0.0302 (13)	0.0432 (16)	−0.0014 (10)	0.0015 (10)	0.0038 (11)
C5	0.0365 (16)	0.0491 (18)	0.0366 (16)	0.0084 (13)	0.0163 (12)	0.0075 (13)
C1	0.0199 (11)	0.0142 (10)	0.0173 (10)	−0.0005 (8)	0.0003 (8)	−0.0053 (8)

### Geometric parameters (Å, º)

**Table:**

Sn1—I2	2.7039 (2)	C13—H13B	0.9800
Sn1—I1	2.7190 (2)	C13—H13C	0.9800
Sn1—O4	2.1164 (15)	C15—H15A	0.9800
Sn1—O8	2.0890 (14)	C15—H15B	0.9800
Sn1—O6	2.1203 (15)	C15—H15C	0.9800
Sn1—O1	2.0826 (15)	C16—H16A	0.9800
O4—C2	1.288 (3)	C16—H16B	0.9800
O8—C4	1.293 (3)	C16—H16C	0.9800
O6—C3	1.286 (3)	N1—H1A	0.9100
O1—C1	1.298 (3)	N1—H1B	0.9100
O3—C2	1.223 (3)	N1—C6	1.510 (3)
C3—C4	1.560 (3)	N1—C8	1.509 (3)
C3—O5	1.221 (3)	C6—H6	1.0000
C4—O7	1.215 (3)	C6—C7	1.513 (3)
C2—C1	1.553 (3)	C6—C5	1.517 (4)
O2—C1	1.217 (3)	C8—H8	1.0000
N2—H2A	0.9100	C8—C10	1.517 (3)
N2—H2B	0.9100	C8—C9	1.518 (4)
N2—C11	1.507 (3)	C10—H10A	0.9800
N2—C14	1.507 (3)	C10—H10B	0.9800
C11—H11	1.0000	C10—H10C	0.9800
C11—C12	1.520 (3)	C9—H9A	0.9800
C11—C13	1.515 (3)	C9—H9B	0.9800
C14—H14	1.0000	C9—H9C	0.9800
C14—C15	1.516 (3)	C7—H7A	0.9800
C14—C16	1.517 (3)	C7—H7B	0.9800
C12—H12A	0.9800	C7—H7C	0.9800
C12—H12B	0.9800	C5—H5A	0.9800
C12—H12C	0.9800	C5—H5B	0.9800
C13—H13A	0.9800	C5—H5C	0.9800
			
I2—Sn1—I1	99.164 (7)	C14—C15—H15A	109.5
O4—Sn1—I2	90.07 (4)	C14—C15—H15B	109.5
O4—Sn1—I1	170.69 (4)	C14—C15—H15C	109.5
O4—Sn1—O6	81.10 (6)	H15A—C15—H15B	109.5
O8—Sn1—I2	91.75 (4)	H15A—C15—H15C	109.5
O8—Sn1—I1	96.37 (4)	H15B—C15—H15C	109.5
O8—Sn1—O4	84.45 (6)	C14—C16—H16A	109.5
O8—Sn1—O6	78.60 (5)	C14—C16—H16B	109.5
O6—Sn1—I2	167.44 (4)	C14—C16—H16C	109.5
O6—Sn1—I1	89.96 (4)	H16A—C16—H16B	109.5
O1—Sn1—I2	101.80 (4)	H16A—C16—H16C	109.5
O1—Sn1—I1	97.85 (4)	H16B—C16—H16C	109.5
O1—Sn1—O4	78.96 (6)	H1A—N1—H1B	107.3
O1—Sn1—O8	158.49 (6)	C6—N1—H1A	108.0
O1—Sn1—O6	85.34 (6)	C6—N1—H1B	108.0
C2—O4—Sn1	113.94 (13)	C8—N1—H1A	108.0
C4—O8—Sn1	115.37 (13)	C8—N1—H1B	108.0
C3—O6—Sn1	115.07 (12)	C8—N1—C6	117.10 (18)
C1—O1—Sn1	114.70 (13)	N1—C6—H6	108.7
O6—C3—C4	115.05 (18)	N1—C6—C7	110.82 (19)
O5—C3—O6	125.98 (19)	N1—C6—C5	107.4 (2)
O5—C3—C4	118.97 (19)	C7—C6—H6	108.7
O8—C4—C3	115.73 (18)	C7—C6—C5	112.3 (2)
O7—C4—O8	124.36 (19)	C5—C6—H6	108.7
O7—C4—C3	119.90 (19)	N1—C8—H8	108.6
O4—C2—C1	115.57 (18)	N1—C8—C10	108.8 (2)
O3—C2—O4	125.0 (2)	N1—C8—C9	109.8 (2)
O3—C2—C1	119.46 (19)	C10—C8—H8	108.6
H2A—N2—H2B	107.2	C10—C8—C9	112.4 (2)
C11—N2—H2A	107.9	C9—C8—H8	108.6
C11—N2—H2B	107.9	C8—C10—H10A	109.5
C11—N2—C14	117.46 (16)	C8—C10—H10B	109.5
C14—N2—H2A	107.9	C8—C10—H10C	109.5
C14—N2—H2B	107.9	H10A—C10—H10B	109.5
N2—C11—H11	109.0	H10A—C10—H10C	109.5
N2—C11—C12	107.79 (17)	H10B—C10—H10C	109.5
N2—C11—C13	109.59 (17)	C8—C9—H9A	109.5
C12—C11—H11	109.0	C8—C9—H9B	109.5
C13—C11—H11	109.0	C8—C9—H9C	109.5
C13—C11—C12	112.3 (2)	H9A—C9—H9B	109.5
N2—C14—H14	108.9	H9A—C9—H9C	109.5
N2—C14—C15	109.93 (18)	H9B—C9—H9C	109.5
N2—C14—C16	107.79 (18)	C6—C7—H7A	109.5
C15—C14—H14	108.9	C6—C7—H7B	109.5
C15—C14—C16	112.3 (2)	C6—C7—H7C	109.5
C16—C14—H14	108.9	H7A—C7—H7B	109.5
C11—C12—H12A	109.5	H7A—C7—H7C	109.5
C11—C12—H12B	109.5	H7B—C7—H7C	109.5
C11—C12—H12C	109.5	C6—C5—H5A	109.5
H12A—C12—H12B	109.5	C6—C5—H5B	109.5
H12A—C12—H12C	109.5	C6—C5—H5C	109.5
H12B—C12—H12C	109.5	H5A—C5—H5B	109.5
C11—C13—H13A	109.5	H5A—C5—H5C	109.5
C11—C13—H13B	109.5	H5B—C5—H5C	109.5
C11—C13—H13C	109.5	O1—C1—C2	115.59 (18)
H13A—C13—H13B	109.5	O2—C1—O1	125.3 (2)
H13A—C13—H13C	109.5	O2—C1—C2	119.15 (19)
H13B—C13—H13C	109.5		
			
Sn1—O4—C2—O3	170.08 (18)	O3—C2—C1—O1	−178.0 (2)
Sn1—O4—C2—C1	−8.8 (2)	O3—C2—C1—O2	1.6 (3)
Sn1—O8—C4—C3	4.8 (2)	O5—C3—C4—O8	175.99 (19)
Sn1—O8—C4—O7	−176.05 (18)	O5—C3—C4—O7	−3.2 (3)
Sn1—O6—C3—C4	0.5 (2)	C11—N2—C14—C15	−57.1 (2)
Sn1—O6—C3—O5	−179.06 (18)	C11—N2—C14—C16	−179.84 (18)
Sn1—O1—C1—C2	7.6 (2)	C14—N2—C11—C12	178.03 (18)
Sn1—O1—C1—O2	−171.97 (18)	C14—N2—C11—C13	−59.5 (2)
O4—C2—C1—O1	1.0 (3)	C6—N1—C8—C10	−166.20 (19)
O4—C2—C1—O2	−179.5 (2)	C6—N1—C8—C9	70.4 (2)
O6—C3—C4—O8	−3.6 (3)	C8—N1—C6—C7	57.7 (3)
O6—C3—C4—O7	177.2 (2)	C8—N1—C6—C5	−179.3 (2)

### Hydrogen-bond geometry (Å, º)

**Table:**

*D*—H···*A*	*D*—H	H···*A*	*D*···*A*	*D*—H···*A*
N1—H1*A*···O2^i^	0.91	2.05	2.909 (2)	156
N1—H1*A*···O3^i^	0.91	2.37	3.006 (3)	127
N1—H1*B*···O3	0.91	2.02	2.916 (2)	168
N2—H2*A*···O7^ii^	0.91	2.07	2.968 (2)	167
N2—H2*B*···O5	0.91	1.96	2.847 (2)	165
N2—H2*B*···O7	0.91	2.48	3.027 (2)	119
C5—H5*C*···O2^i^	0.98	2.60	3.359 (3)	135
C13—H13*B*···O2^iii^	0.98	2.45	3.397 (3)	162
C14—H14···O5^iv^	1.00	2.52	3.508 (3)	170

Symmetry codes: (i) −*x*+1, −*y*+1, −*z*; (ii) −*x*+2, −*y*+1, −*z*+1; (iii) −*x*+1, *y*+1/2, −*z*+1/2; (iv) −*x*+1, −*y*+1, −*z*+1.
