# Supplementary figures and images for: Crystal structure of bis­(diiso­propyl­ammonium) cis-di­iodido­bis­(oxolato-κ2 O 1,O 2)stannate(IV)
Source: Acta Crystallogr E Crystallogr Commun. 2018 Mar 9;74(Pt 4):502–4. doi: 10.1107/S2056989018003602 (PMC5946977; doi:10.1107/S2056989018003602)

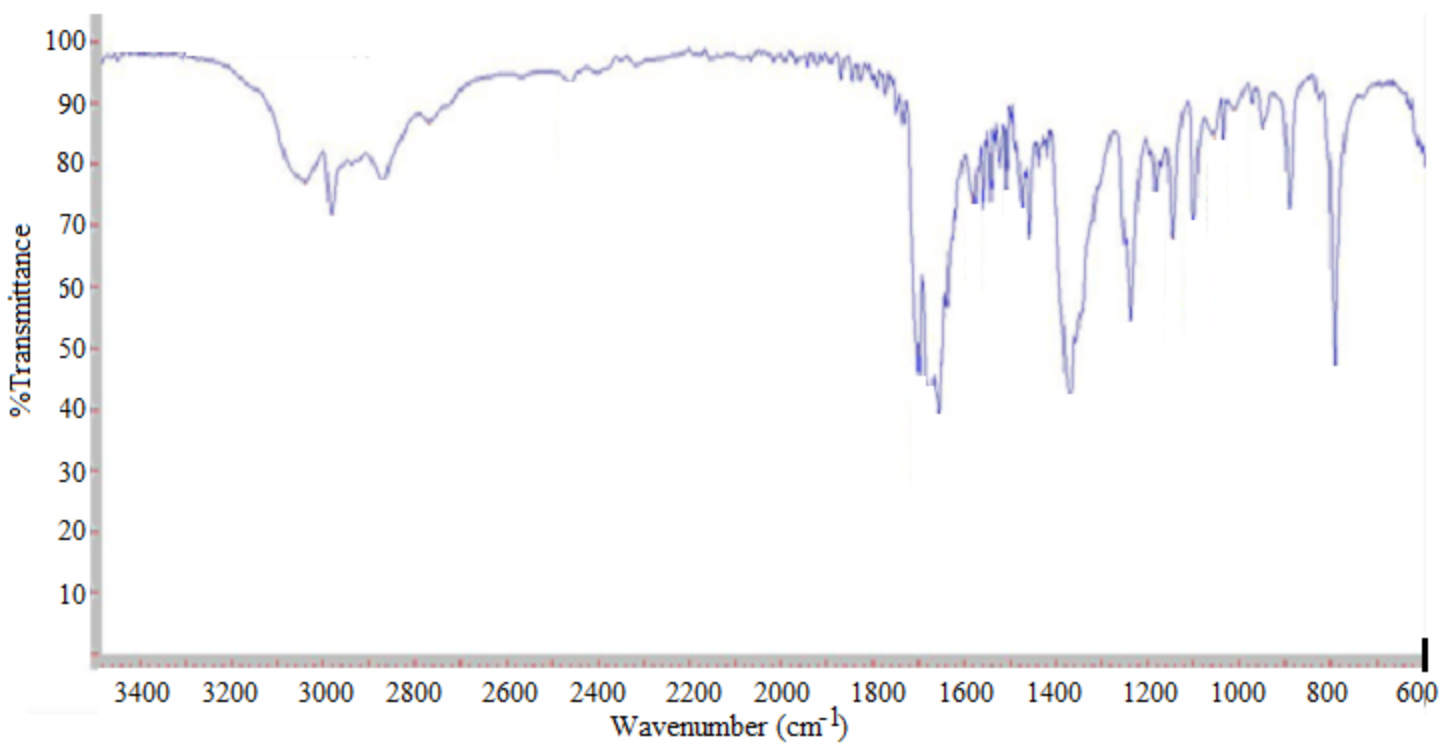

Supplement: Supplementary file 3 [file e-74-00502-sup3.pdf]
